# Supplementary material for: The structural insight into the functional modulation of human anion exchanger 3
Source: Nat Commun. 2024 Jul 20;15:6134. doi: 10.1038/s41467-024-50572-x (PMC11271275; doi:10.1038/s41467-024-50572-x)
Supplement: Supplementary file 1 — Supplementary Information [file 41467_2024_50572_MOESM1_ESM.pdf]

Supplementary information for

The structural insight into the functional modulation of human anion exchanger 3

Liyan Jian[1,2 †], Qing Zhang[2,3†], Deqiang Yao[2,4†], Qian Wang[2], Moxin Chen[5,6], Ying Xia[2], Shaobai Li[2], Yafeng Shen[2], Mi Cao[2], An Qin[1,7*], Lin Li[5,6*], Yu Cao[1,2*]

Corresponding author. Email: yu.cao@shsmu.edu.cn (Y. C.); lin_li@sjtu.edu.cn (L. L.); dr_qinan@163.com (A. Q.)

Supplementary items:
Supplementary Figs. 1-13
Supplementary Table 1-2

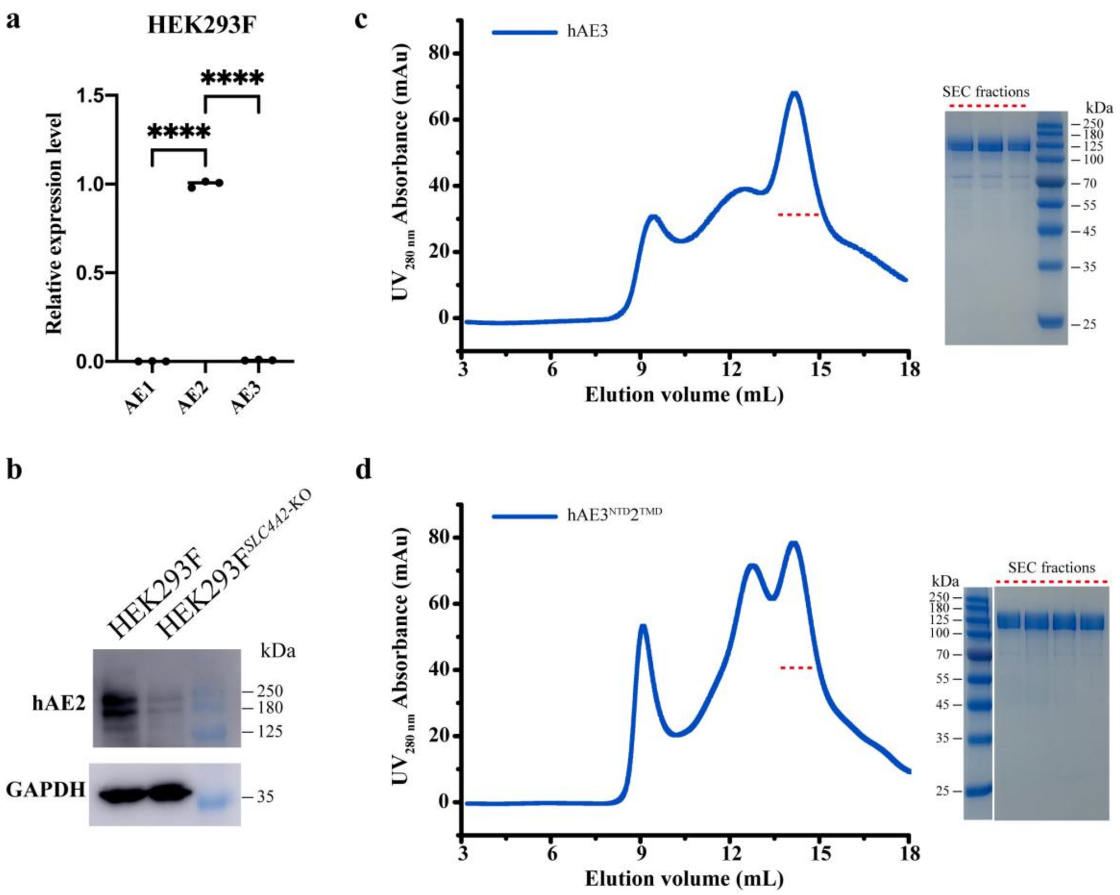

Supplementary Fig. 1. The gene knockout of human *SLC4A2* and the purification of the hAE3 for cryo-EM.

a, The qPCR quantitation on the expression levels of AE1-3 in HEK293F cells, with all experiments replicated independently three times to ensure consistency. One-way ANOVA with Tukey's multiple comparisons test was performed, ****: p<0.0001. **b,** Western blot analysis of the endogenous hAE2 expression in HEK293F *SLC4A2*-KO cell line, with all experiments replicated independently three times to ensure consistency. **c,** The purification of $hAE3^{300\text{-}1232}$. **d,** The purification of $hAE3^{NTD}2^{TMD}$ chimera. Source data are provided as a Source Data file.

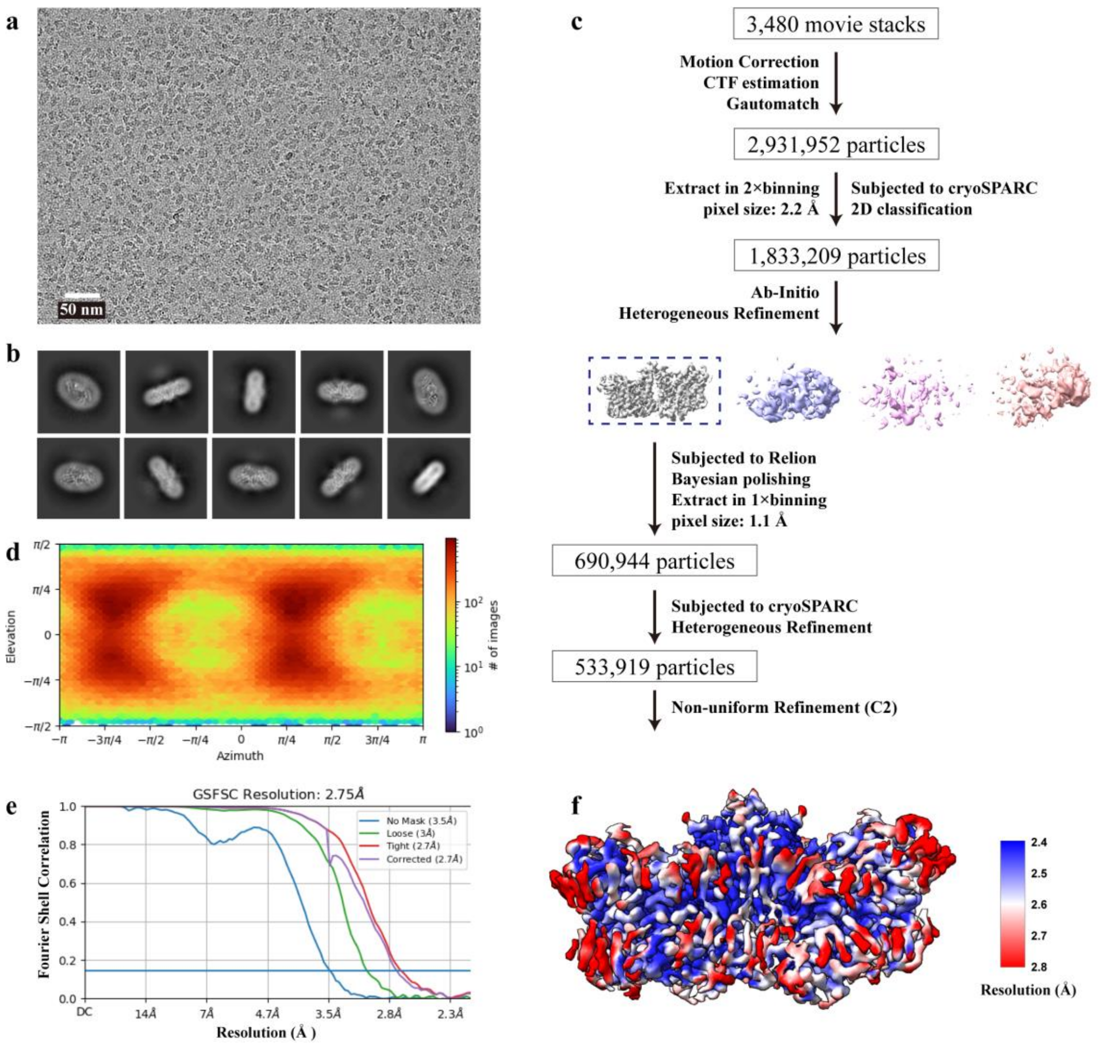

Supplementary Fig. 2. The cryo-EM analysis of hAE3$^{HCO_3^-}$.

a, A representative micrograph of hAE3$^{HCO_3^-}$. Most of the micrographs were similarly of high quality. **b,** A representative 2D class average. **c,** The flow chart of cryo-EM data processing on hAE3$^{HCO_3^-}$. **d,** Orientation distribution of particles for the final map reconstruction. **e,** The gold-standard Fourier shell correlation (FSC) curves for the final cryo-EM map of hAE3$^{HCO_3^-}$, generated by cryoSPARC with non-uniform refinement. Source data are provided as a Source Data file. **f,** Local-resolution map of hAE3$^{HCO_3^-}$.

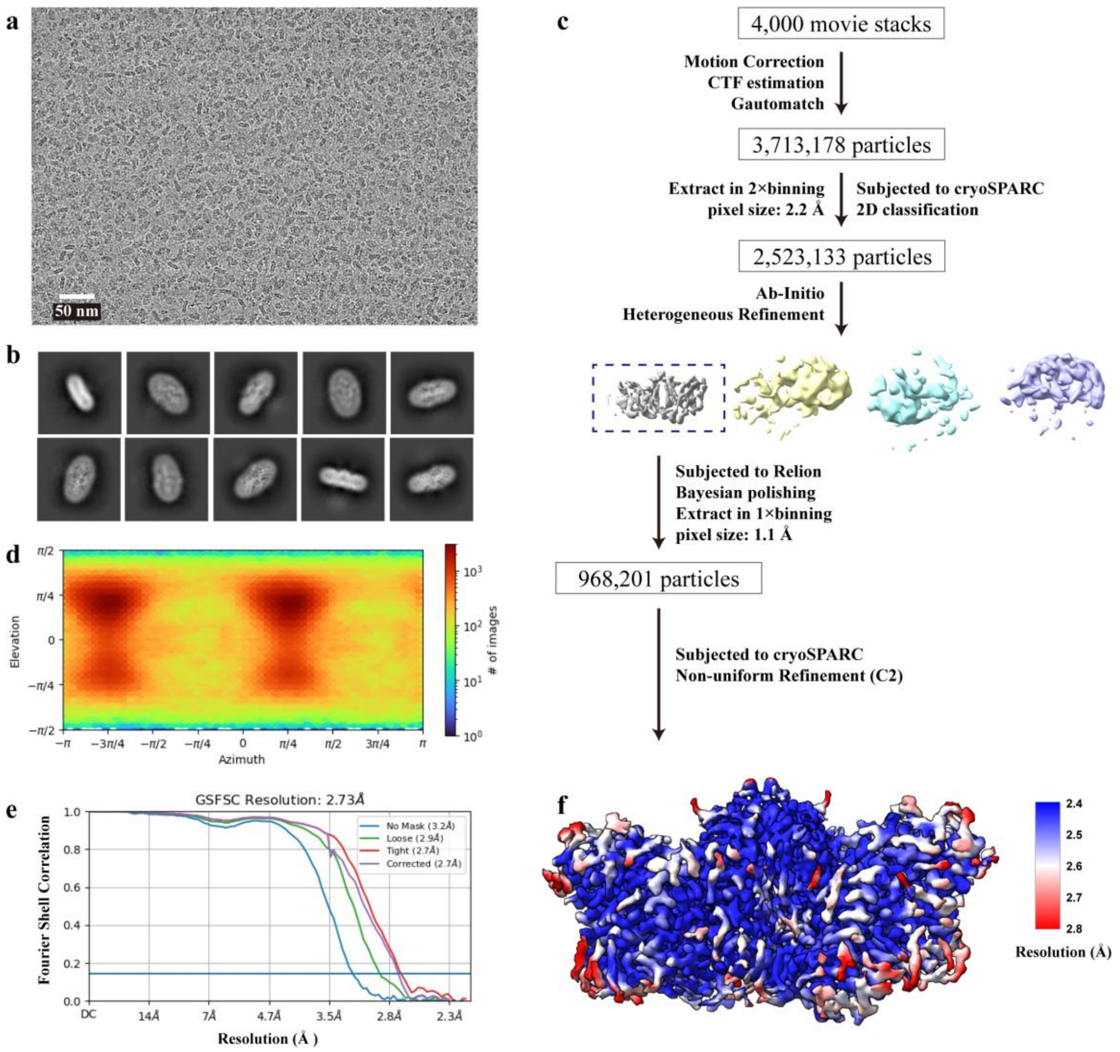

Supplementary Fig. 3. The cryo-EM analysis of hAE3$^{HCO_3^-/DIDS}$.

a, A representative micrograph of hAE3$^{HCO_3^-/DIDS}$. Most of the micrographs were similarly of high quality. **b,** A representative 2D class average. **c,** The flow chart of cryo-EM data processing on hAE3$^{HCO_3^-/DIDS}$. **d,** Orientation distribution of particles for the final map reconstruction. **e,** The gold-standard Fourier shell correlation (FSC) curves for the final cryo-EM map of hAE3$^{HCO_3^-/DIDS}$, generated by cryoSPARC with non-uniform refinement. Source data are provided as a Source Data file. **f,** Local-resolution map of hAE3$^{HCO_3^-/DIDS}$.

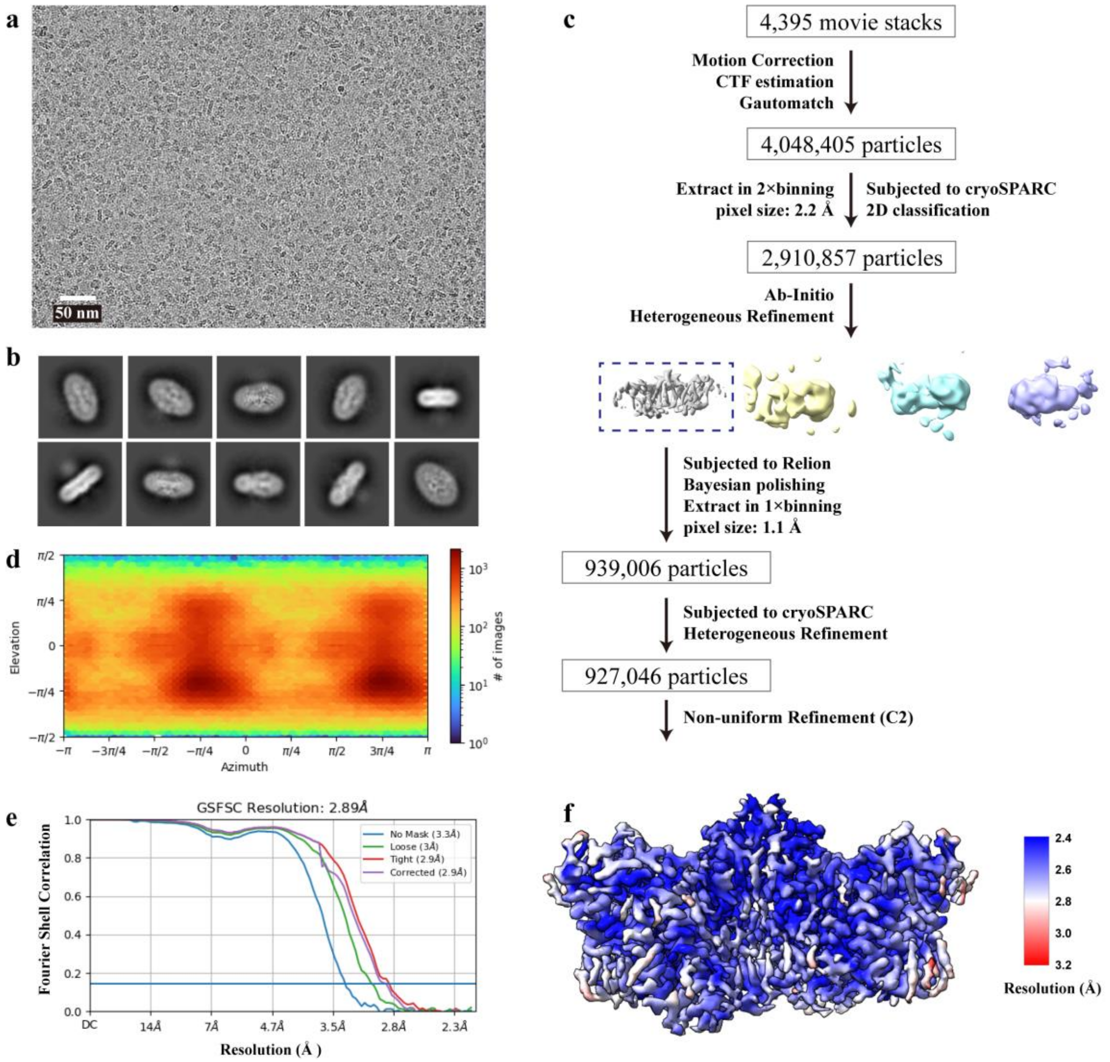

Supplementary Fig. 4. The cryo-EM analysis of hAE3 apo.

a, A representative micrograph of hAE3 apo. Most of the micrographs were similarly of high quality. **b,** A representative 2D class average. **c,** The flow chart of cryo-EM data processing on hAE3 apo. **d,** Orientation distribution of particles for the final map reconstruction. **e,** The gold-standard Fourier shell correlation (FSC) curves for the final cryo-EM map of hAE3 apo, generated by cryoSPARC with non-uniform refinement. Source data are provided as a Source Data file. **f,** Local-resolution map of hAE3 apo.

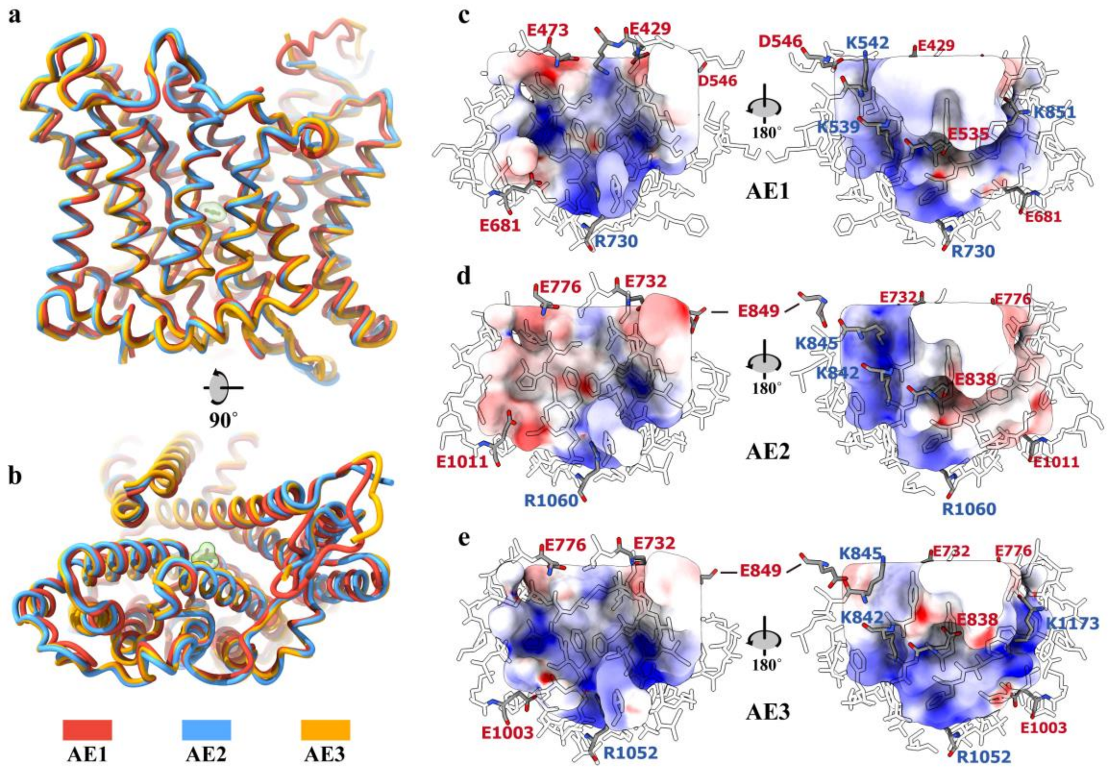

Supplementary Fig. 5. The comparison among the structure of the transmembrane domains of human AE1-3 in the outward-facing conformations.

a, and b, The TMDs of human AE1-3 were superposed as cartoon models and viewed parallel to the cell membrane (a) and from the extracellular side (b). The structural models of AE1 and AE2 were generated from coordinate fils with PDB IDs 4YZF (for AE1, in red) and 8GVF (for AE2, in blue). **c-e,** The outer vestibules identified from the AE1-3 in the outward-facing conformations. The outer vestibules were determined with Hollow and shown as the solvent-accessible electrostatic surface–potential maps. The residues surrounding the outer vestibules were shown as stick models, and those with electric charges were highlighted with labels.

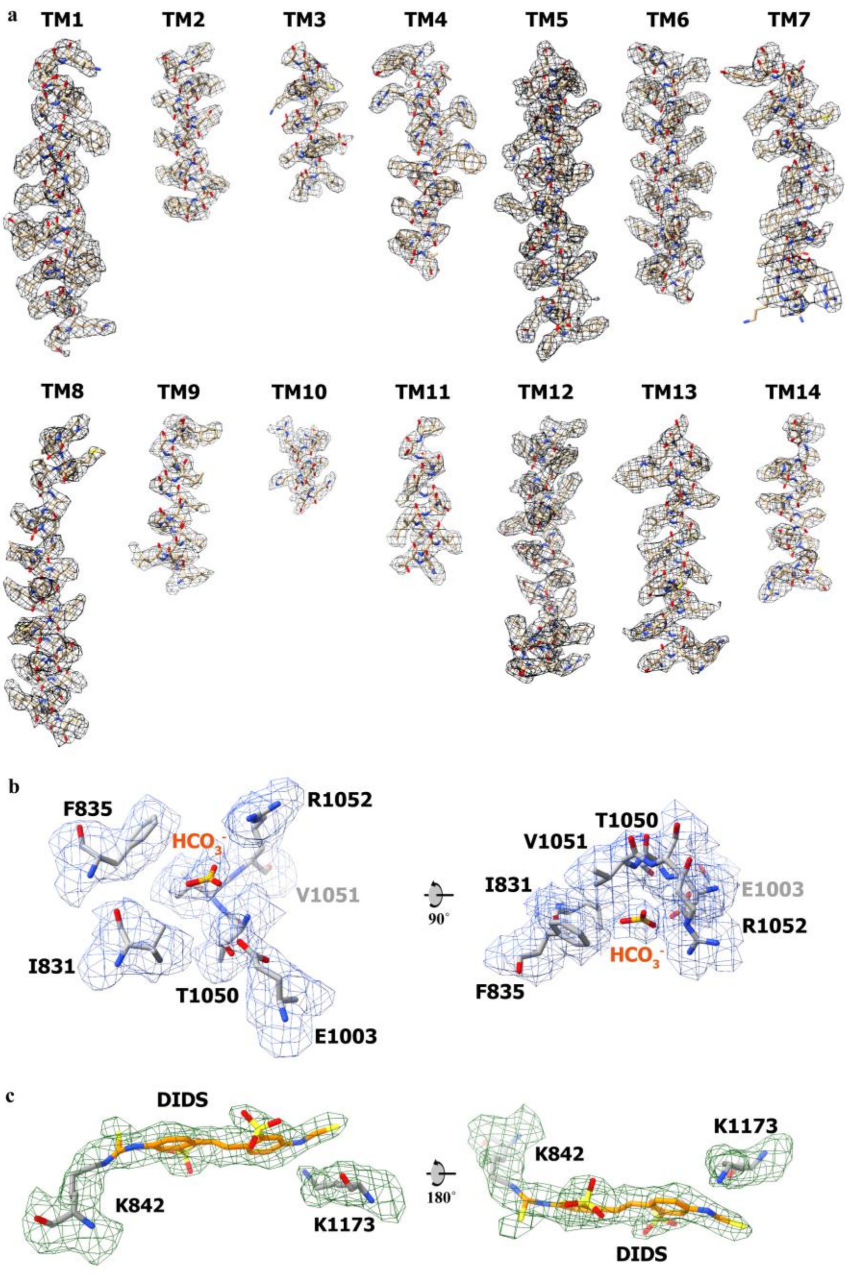

Supplementary Fig. 6. The cryo-EM density map and the model-fitting for the key secondary structures of hAE3.

a, The helices TMH1-12 of $hAE3^{HCO3^-}$ were shown combined with their corresponding cryo-EM density map. **b,** The density maps for HCO_3^- bound in $hAE3^{HCO3^-}$ structure are shown at the same contour level as residues I831, F835, E1003, T1050, and R1052. **c,** The density maps for DIDS from $hAE3^{HCO3^-/DIDS}$ structure are shown at the same contour level as the residues K842 and K1173.

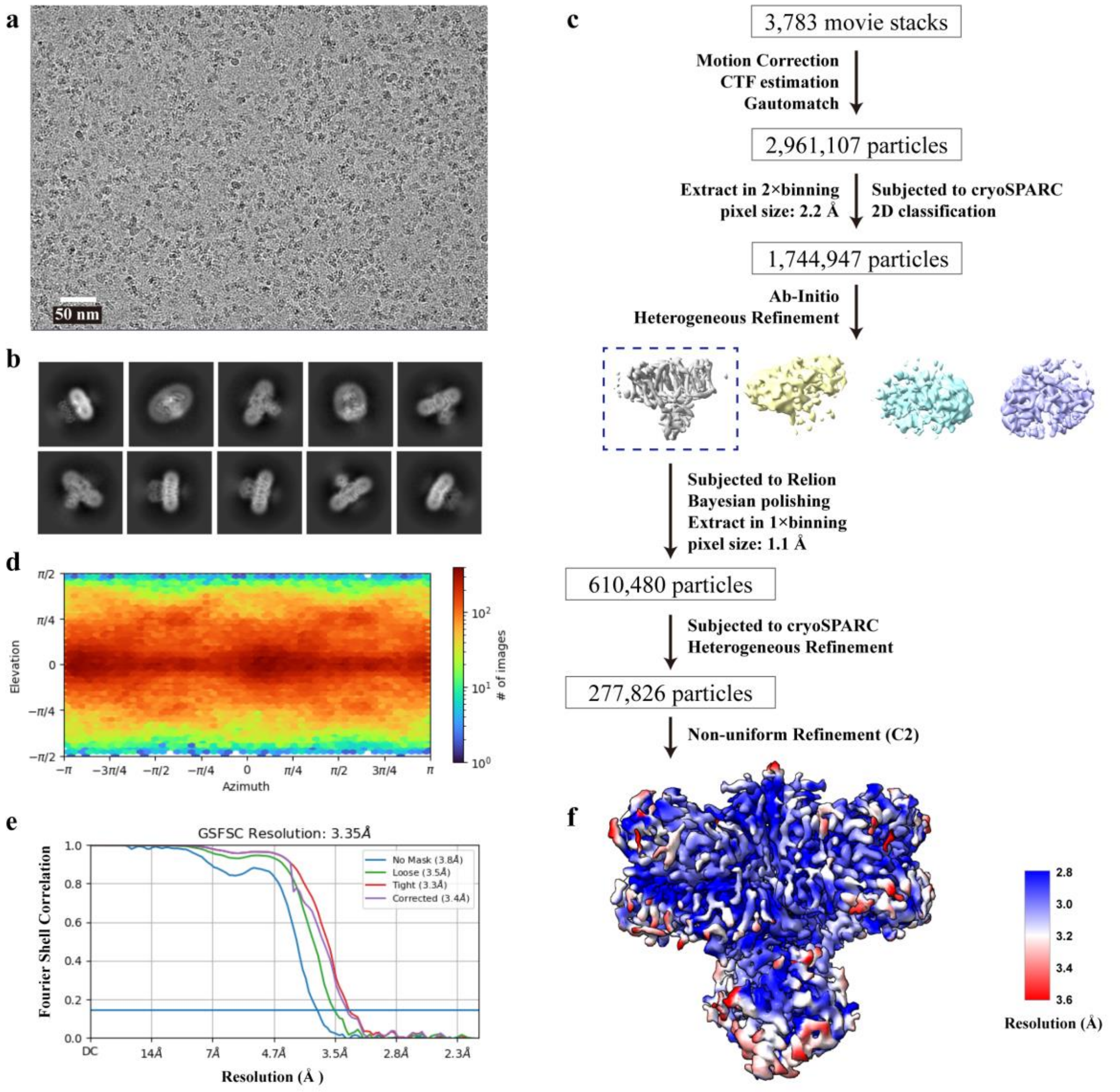

Supplementary Fig. 7. The cryo-EM analysis of hAE3^{NTD}2^{TMD}.

a, A representative micrograph of hAE3^{NTD}2^{TMD}. Most of the micrographs were similarly of high quality. **b,** A representative 2D class average. **c,** The flow chart of cryo-EM data processing on hAE3^{NTD}2^{TMD}. **d,** Orientation distribution of particles for the final map reconstruction. **e,** The gold-standard Fourier shell correlation (FSC) curves for the final cryo-EM map of hAE3^{NTD}2^{TMD}, generated by cryoSPARC with non-uniform refinement. Source data are provided as a Source Data file. **f,** Local-resolution map of hAE3^{NTD}2^{TMD}.

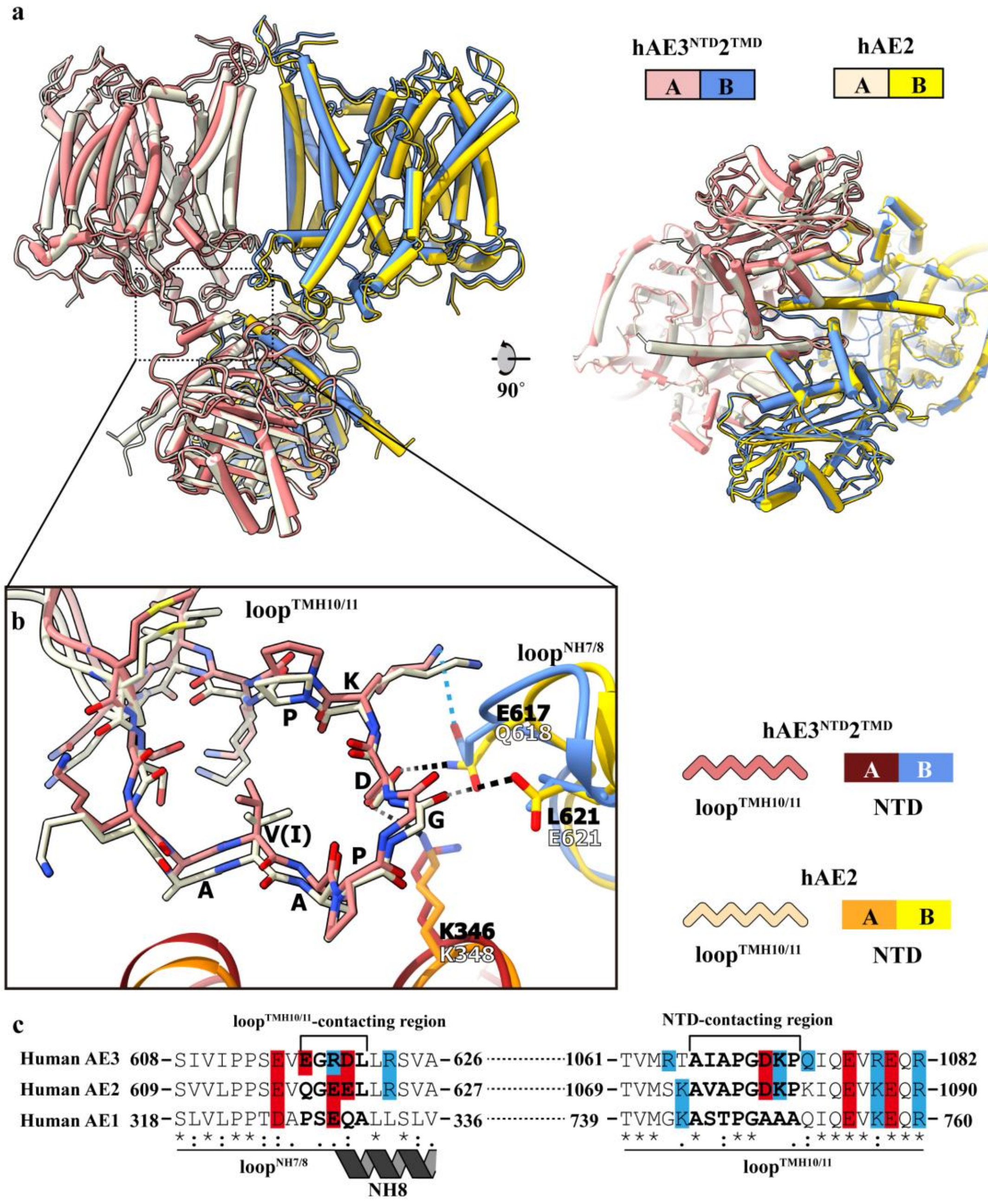

Supplementary Fig. 8. The cryo-EM structure of hAE3^{NTD}2^{TMD} chimera.

a, The structural superposition of full-length structures of human AE2 and AE3^{NTD}2^{TMD} chimera. The human AE2 and AE3^{NTD}2^{TMD} chimera were shown as cartoon models and viewed parallel to the cell membrane (left) and from the cytoplasmic side (right). The structural models of hAE2 were generated from a coordinate file with PDB ID 8GVH. **b,** The comparison between the NTD-TMD interactions in hAE2 and AE3^{NTD}2^{TMD} chimera. The dotted frame in **a** was enlarged, where the loop$^{TMH10/11}$ from hAE2 and AE3^{NTD}2^{TMD} were shown as stick models colored by elements. The NTDs from hAE2 and AE3^{NTD}2^{TMD} were shown as cartoon models with the contacting residue as stick models. The models were colored to the scheme shown on the right. **c,** A protein sequence alignment between the contacting regions of NTD-TMD interfaces from human AE3，AE2 and AE1.

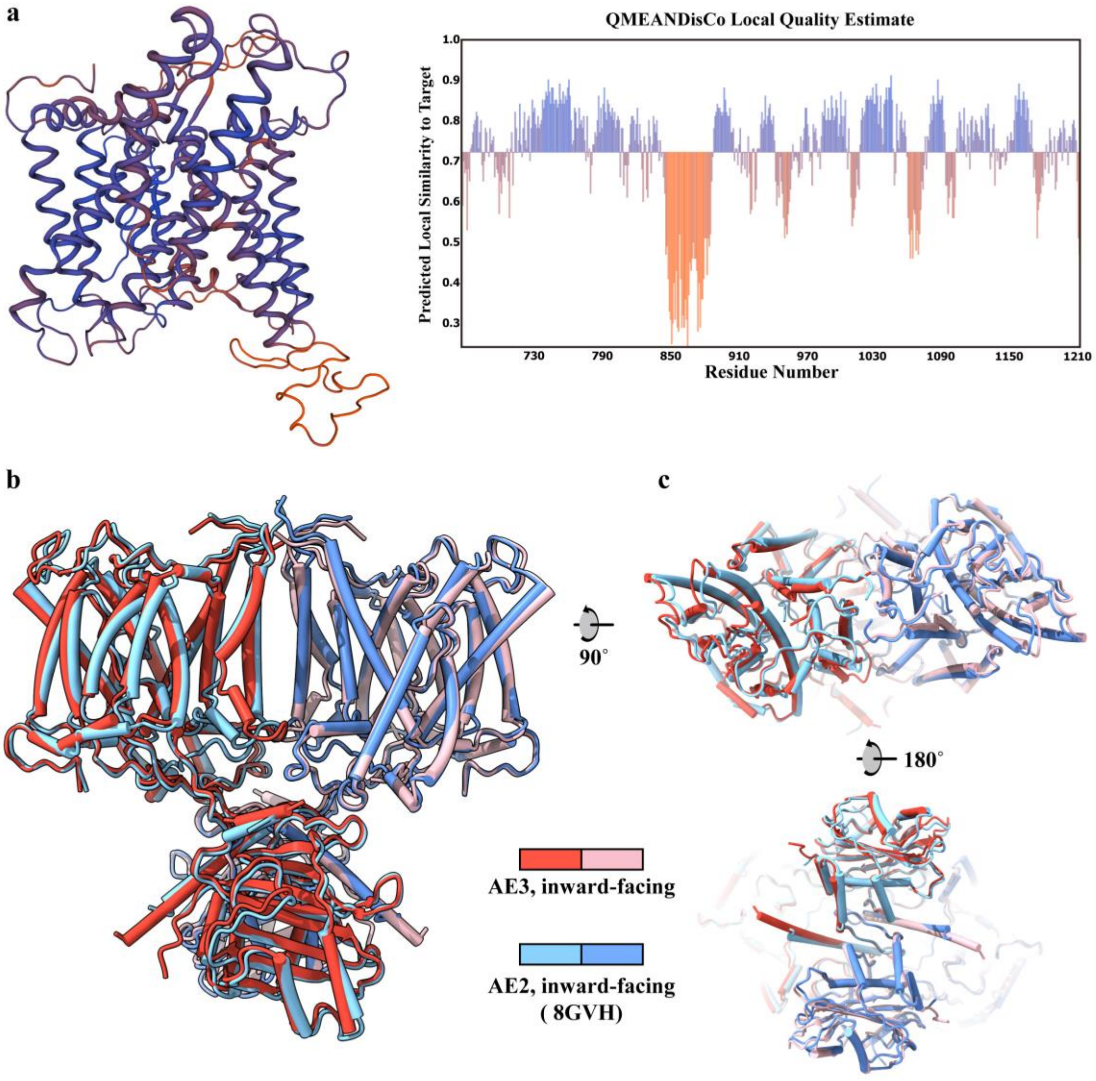

Supplementary Fig. 9. The homology-modeling of human AE3 in its inward-facing conformation.

a, The structure of human AE3 TMD as computed using the SWISS-MODEL server homology modeling pipeline. Left: The predicted model was shown as a cartoon model and colored by the QMEANDisCo local score for each residue (orange to blue scores for low to high). Right: The QMEANDisCo local score vs. residue plot. Source data are provided as a Source Data file. **b and c,** The structural superposition between AE2 and AE3 in their inward-facing conformations. The structural models of hAE2 were generated from a coordinate file with PDB ID 8GVH. The structural models of hAE3 were generated by superposing the predicted AE3 TMD in inward-facing conformation with hAE3^{NTD}2^{TMD}, followed by merging the AE3 TMD and NTD.

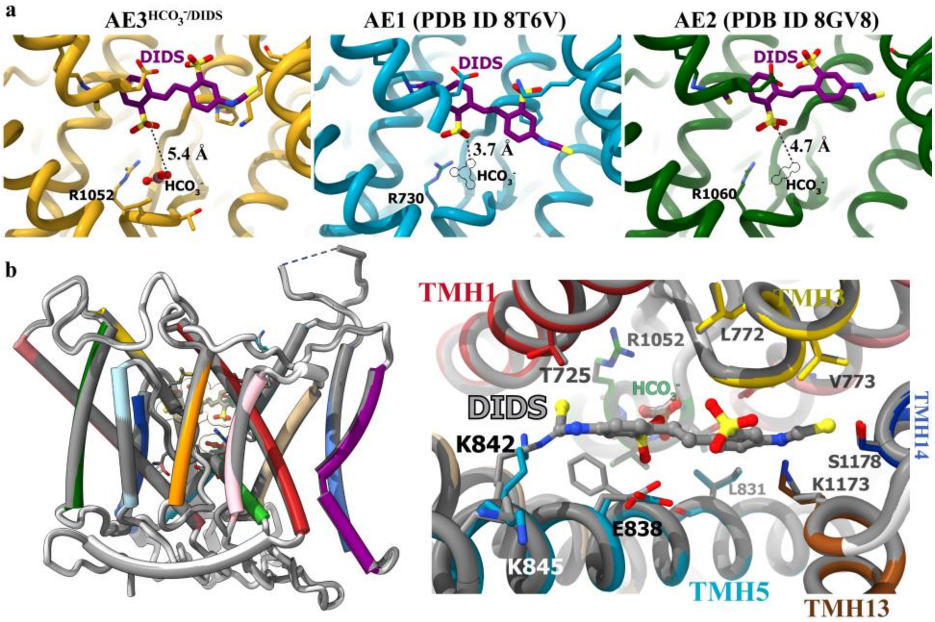

Supplementary Fig. 10. The structural comparison among DIDS bound in AEs.

a, The DIDS bound in the outer vestibules of AE3 (left), AE1 (middle), and AE2 (right). The AE1-3 structures were shown as cartoon model, with the DIDS and HCO3- shown as stick models. The HCO_3^- binding sites in AE1 and 2 were indicated with transparent stick model. **b,** The structural superposition between hAE3$^{HCO_3^-}$ and hAE3$^{HCO_3^-/DIDS}$**.** Left: the structural superposition between hAE3$^{HCO_3^-}$ and hAE3$^{HCO_3^-/DIDS}$. Both structures were shown as cartoon model, with hAE3$^{HCO_3^-}$ colored by helices in a rainbow gradient and hAE3$^{HCO_3^-/DIDS}$ colored in gray. The HCO_3^- and DIDS were shown as stick model. Right: Enlarged view of the binding pocket of DIDS in the structural superposition between hAE3$^{HCO_3^-}$ and hAE3$^{HCO_3^-/DIDS}$. The key interacting residues were shown as stick model and colored as their respective backbone.

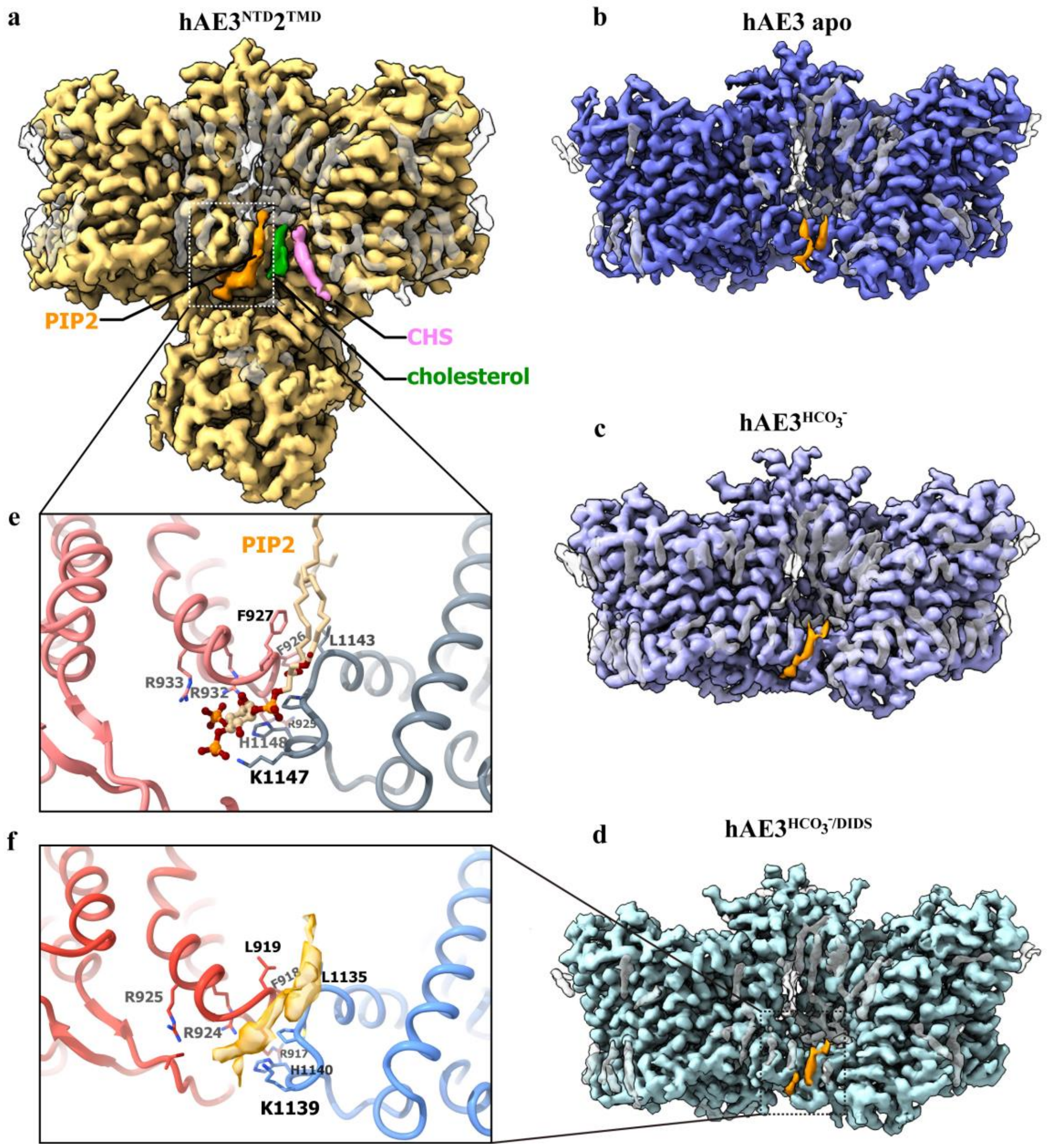

Supplementary Fig. 11. The lipids bound in hAE3^{NTD}2^{TMD} and hAE3.

a-d, The non-proteinous densities identified in the outer surfaces of the TMDs from AE2 and AE3 structures. The protein density maps were shown for hAE3^{NTD}2^{TMD} (**a**), hAE3 apo (**b**), hAE3$^{HCO_3^-}$ (**c**), and hAE3$^{HCO_3^-/DIDS}$ (**d**), with the non-proteinous densities highlighted in orange for PIP2, green for cholesterol, pink for CHS, and transparent gray for other unidentified lipids. **e and f,** The enlarged view of the PIP$_2$ binding sites in hAE3^{NTD}2^{TMD} (**e**) and hAE3$^{HCO_3^-/DIDS}$ (**f**). The TMDs of AE2 and AE3 were shown as cartoon model. The PIP$_2$ molecule and the contacting residues were shown as stick models. The non-proteinous densities in AE3 were shown as an orange surface model and set to transparent.

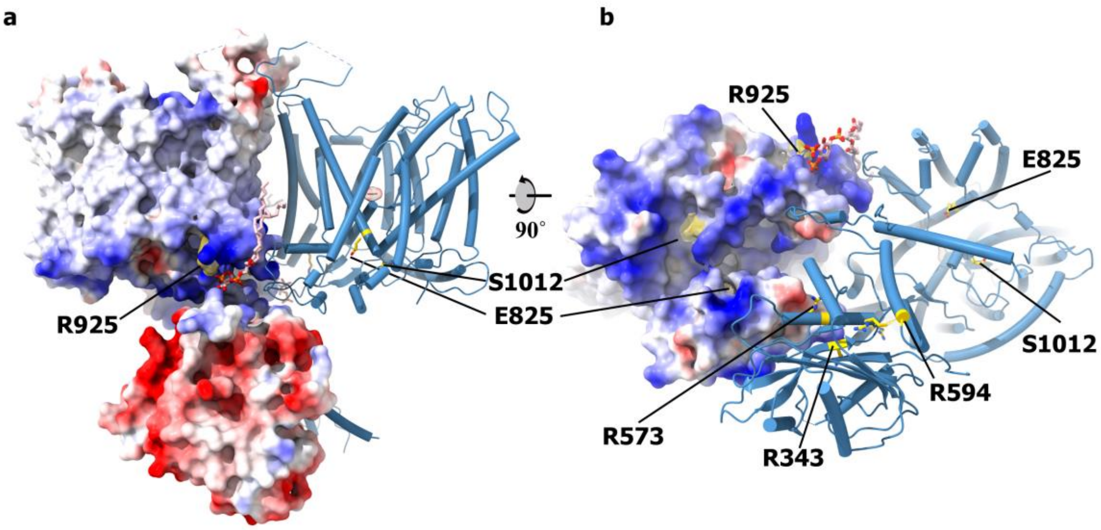

Supplementary Fig. 12. The locations of the pathogenic mutation sites in AE3.

a and b, The full-length AE3 in its inward-facing conformation model was generated by merging the TMD of $hAE3^{HCO_3^-}$ model and the NTDs/PIP_2 of the $hAE3^{NTD}2^{TMD}$ model. Protomer A was shown as solvent-accessible electrostatic surface–potential maps, and protomer B was shown as a cartoon model. The pathogenic mutation sites were highlighted in yellow. The PIP_2 was shown as a stick model colored in pink.

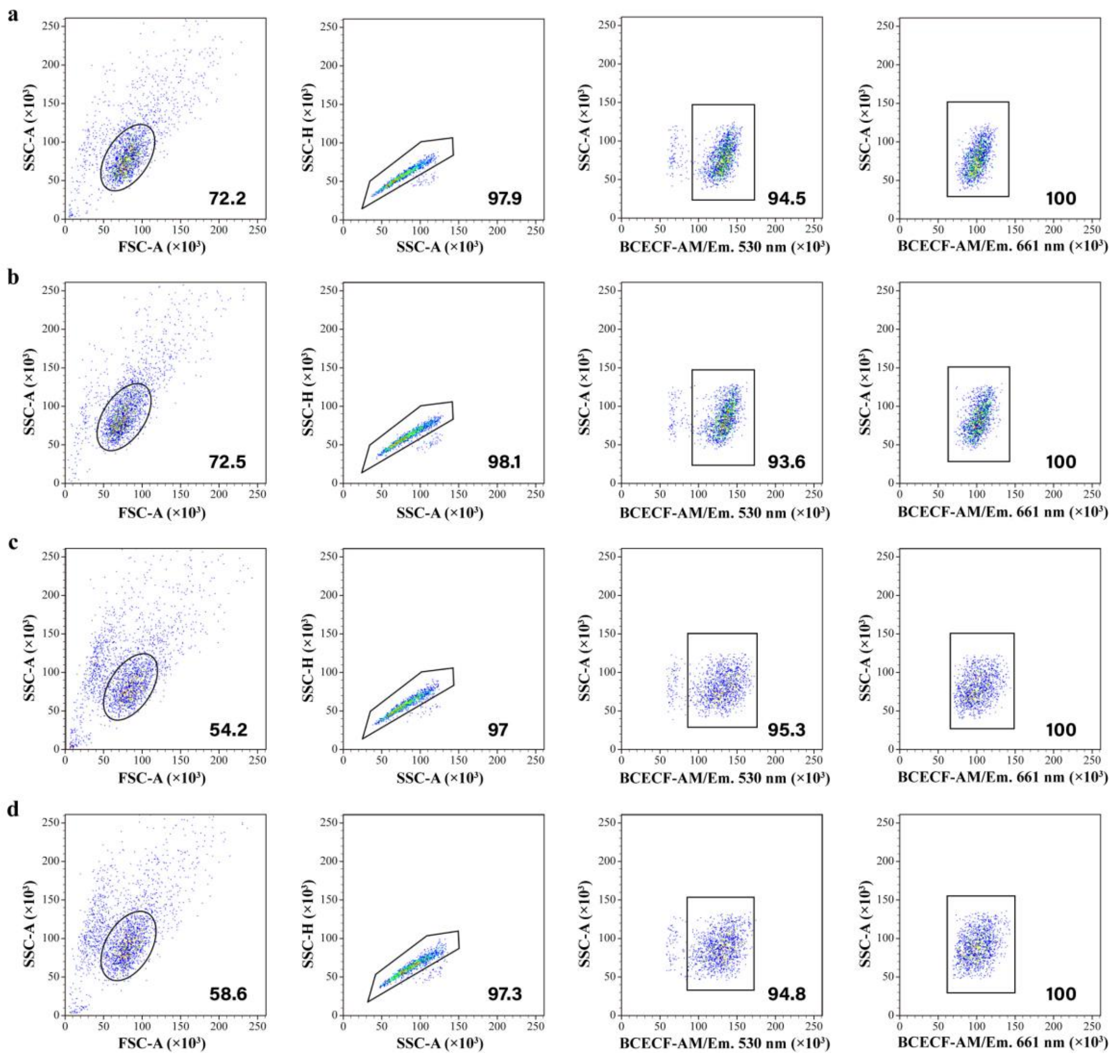

Supplementary Fig. 13. The sorting strategies used in FACS for the anion exchange activity assay.

a, Untransfected *SLC4A2*-KO HEK293F cells in a Cl^--free buffer were used to set the gates. Forward Scatter (FSC-A) vs. Side Scatter (SSC-A) plot was used to remove most of the debris, air bubbles, and laser noise in the first step. Doublets and multiplets were excluded using SSC-A vs. SSC-H plots. Only cells positive for 488 nm excitation with both 530 nm emission and 661 nm emission were included. **b,** Untransfected *SLC4A2*-KO HEK293F cells in a Cl^--containing buffer. **c,** *SLC4A2*-KO HEK293F cells transfected with hAE3 expression vector in a Cl^--free buffer. **d,** *SLC4A2*-KO HEK293F cells transfected with hAE3 expression vector in a Cl^--containing buffer.

Supplementary table 1. Summary of cryo-EM data collection, processing, and structure refinement.

	AE3 apo (EMDB-39050) (PDB ID 8Y8K)	hAE3$^{HCO_3^-}$ (EMDB-39035) (PDB ID 8Y86)	hAE3$^{HCO_3^-/DIDS}$ (EMDB-39034) (PDB ID 8Y85)	hAE3^{NTD}2^{TMD} (EMDB-60225) (PDB ID 8ZLE)
**Data collection and processing**				
Magnification	81000	81000	81000	81000
Voltage (kV)	300	300	300	300
Electron exposure	50	50	50	50
Defocus range	1.2-3.1	1.1-3.0	1.2-2.8	1.5-3.0
Pixel size	1.1	1.1	1.1	1.1
Symmetry	C2	C2	C2	C2
Initial particle	4,048,405	2,931,952	3,713,178	2,961,107
Final particle	927,046	533,919	968,201	277,826
Map resolution (Å)	2.89	2.75	2.73	3.35
FSC threshold	0.143	0.143	0.143	0.143
**Refinement**				
Initial model used	8GVC	8Y8K	8Y8K	8GVH
Model resolution (Å)	2.89 (0.143) 3.26 (0.5)	2.89 (0.143) 3.24 (0.5)	2.89 (0.143) 3.24 (0.5)	3.32 (0.143) 3.79 (0.5)
**Model composition**				
Non-hydrogen	8052	8060	8116	12666
Protein residues	1028	1028	1028	1562
Ligands	--	BCT: 2	BCT: 2; 4KU: 2	PT5: 2; CLR: 2; Y01: 2
**B factors (Å^2)**				
Protein	51.36	100.98	50.20	81.38
Ligand	--	114.21	57.45	101.59
**R.m.s. deviations**				
Bond length (Å)	0.003	0.004	0.002	0.003
Bond angles (°)	0.491	0.535	0.503	0.532
**Validation**				
MolProbity score	1.68	1.12	1.33	1.84
Clashscore	2.24	2.36	2.89	5.34
Rotamer outliers	1.37	1.14	1.37	2.21
**Ramachandran plot**				
Favored (%)	97.25	97.75	97.16	95.73
Allowed (%)	2.75	2.25	2.84	4.27
Outlier (%)	0	0	0	0

Supplementary table 2. Primer List

hAE2-F	CCGGCGCGCCATGGGGAGGAAGACTTTGAGTACCACC
hAE2-R	CCGCGGCCGCCTACACAGGCATGGGCA
hAE3-F	CCGGCGCGCCATGCCCGCCGGCCTGGCCCCCATCC
hAE3-R	CCGCGGCCGCCTACACTGGCATGTGCAGCTCATTG
AE3-F767A-F	GTGGTTGGCGCCTCTGGGCCGCTGCTTGTGTTTGAG
AE3-F767A-R	GGCCCAGAGGCGCCAACCACAAGCAGC
AE3-S768A-F	GGTTGGCTTCGCTGGGCCGCTGCTTGTGTTTGAG
AE3-S768A-R	CGGCCCAGCGAAGCCAACCACAAGCAGC
AE3-I831A-F	CTTTGCCTTTCTCGCCTCACTCATTTTCATCTACGAGAC
AE3-I831A-R	GAAAATGAGTGAGGCGAGAAAGGCAAAGATCTCCTGG
AE3-F835A-F	CATCTCACTCATTGCCATCTACGAGACCTTCTACAAGC
AE3-F835A-R	GTCTCGTAGATGGCAATGAGTGAGATGAGAAAGGCAAAG
AE3-E1003A-F	CTGATCTTCATGGCGACACAGATCACGGCGCTTATC
AE3-E1003A-R	GTGATCTGTGTCGCCATGAAGATCAGGATGAGGAC
AE3-T1050A-F	CGGCTGCCGCGGTCCGCTCCGTC
AE3-T1050A-R	GCGGACCGCGGCAGCCGTGAGCC
AE3-V1051A-F	CTGCCACGGCCCGCTCCGTCACCC
AE3-V1051A-R	CGGAGCGGGCCGTGGCAGCCGTG
AE3-R1052A-F	GCCACGGTCGCCTCCGTCACCCATGTC
AE3-R1052A-R	GGTGACGGAGGCGACCGTGGCAGCCGTG
AE3-NTD-R	GCCGAAGGGGATCATCTTCAGGGGTTGCCTCAG
AE2-TMD-F	GAGGCAACCCCTGAAGATGATCCCCTTCGGC
AE2-sgRNA-F	CACCGTCTTGCGGCGGCGTGCATCCGG
AE2-sgRNA-R	AAACCCGGATGCACGCCGCCGCAAGAC
Actin-F	CACCATTGGCAATGAGCGGTTC
Actin-R	AGGTCTTTGCGGATGTCCACGT
SLC4A1-F	CGAAGGCGCTATCAGTCCAG
SLC4A1-R	TGATGTCACTCAGGTAATAGGGG
SLC4A2-F	ACCCTCATGTCAGACAAGCAA
SLC4A2-R	TCCTCTCGCTTCTTGAGCATC
SLC4A3-F	GCCCATCGGCCAGTTATGAC
SLC4A3-R	AGGGCTTTTTCACTAAGTGTCG
